# CD8 T Cell Responses to an Immunodominant Epitope within the Nonstructural Protein NS1 Provide Wide Immunoprotection against Bluetongue Virus in IFNAR^−/−^ Mice

**DOI:** 10.1128/JVI.00938-18

**Published:** 2018-07-31

**Authors:** Alejandro Marín-López, Eva Calvo-Pinilla, Diego Barriales, Gema Lorenzo, Alejandro Brun, Juan Anguita, Javier Ortego

**Affiliations:** a Center for Animal Health Research, INIA-CISA, Valdeolmos, Madrid, Spain; b Macrophage and Tick Vaccine Lab, CIC bioGUNE, Bizkaia Technology Park, Derio, Bizkaia, Spain; c Ikerbasque, Basque Foundation for Science, Bilbao, Bizkaia, Spain; Instituto de Biotecnologia/UNAM

**Keywords:** bluetongue, NS1, MVA, multiserotype, DIVA, vaccine, CD8 T cell response

## Abstract

The development of vaccines against bluetongue, a prevalent livestock disease, has been focused on surface antigens that induce strong neutralizing antibody responses. Because of their antigenic variability, these vaccines are usually serotype restricted. We now show that a single highly conserved nonstructural protein, NS1, expressed in a modified vaccinia Ankara virus (MVA) vector can provide multiserotype protection in IFNAR^−/−^ 129 mice against bluetongue virus (BTV) that is largely dependent on CD8 T cell responses. We found that the protective antigenic capacity of NS1 resides within the N terminus of the protein and is provided in the absence of neutralizing antibodies. The protective CD8 T cell response requires the presence of a specific peptide within the N terminus of NS1, since its deletion ablates the efficacy of the vaccine formulation. These data reveal the importance of the nonstructural protein NS1 in CD8 T cell-mediated protection against multiple BTV serotypes when vectorized as a recombinant MVA vaccine.

**IMPORTANCE** Conventional vaccines have controlled or limited BTV expansion in the past, but they cannot address the need for cross-protection among serotypes and do not allow distinguishing between infected and vaccinated animals (DIVA strategy). There is a need to develop universal vaccines that induce effective protection against multiple BTV serotypes. In this work we have shown the importance of the nonstructural protein NS1, conserved among all the BTV serotypes, in CD8 T cell-mediated protection against multiple BTV serotypes when vectorized as a recombinant MVA vaccine.

## Abstract (precis)

Conventional vaccines have controlled or limited BTV expansion in the past, but they cannot address the need for cross-protection among serotypes and do not allow distinguishing between infected and vaccinated animals (DIVA strategy). There is a need to develop universal vaccines that induce effective protection against multiple BTV serotypes. In this work we have shown the importance of the nonstructural protein NS1, conserved among all the BTV serotypes, in CD8 T cell-mediated protection against multiple BTV serotypes when vectorized as a recombinant MVA vaccine.

## INTRODUCTION

Bluetongue virus (BTV) causes a hemorrhagic disease of ruminants that is transmitted by Culicoides species of biting midges. To date, 27 serotypes of BTV have been identified (1), with two more putative serotypes and several other variants being further described (2–6). BTV has been historically prevalent in tropical and subtropical regions located between 35° S and 45° N, coinciding with the presence of competent Culicoides vectors (7). However, outbreaks have been reported farther north, including in several countries in Europe, Asia, Oceania, and the Americas. Since 1998, BTV serotypes 1, 2, 4, 6, 8, 9, 11, and 16 have been introduced in Europe, while additional novel serotypes have recently invaded historic countries of endemicity such as Israel, Australia, and the United States. Five BTV serotypes have long been recognized as enzootic in North America. Since 1998, 10 additional previously exotic serotypes have been isolated in the southeastern United States, and BTV infection of sheep was detected for the first time in Ontario, Canada, in 2015, which represents the farthest North expansion of BTV in North America (5). Worldwide, BTV has been estimated to cause direct (disease) and indirect (trade, vaccines, etc.) losses of over $3 billion per year (8, 9).

The development of an effective vaccine remains an important goal for the safe and cost-effective control of this disease. Bluetongue vaccine development has classically focused on inactivated and attenuated virus. However, live attenuated viral vaccines are associated with clinical signs, viremia compatible with transmission, and risk of gene segment reassortment (10, 11). Moreover, these vaccines are serotype specific, inducing neutralizing antibodies against the outer capsid protein VP2. Although conventional vaccines have controlled or limited BTV expansion in the past, they cannot address the need for cross-protection among serotypes and do not allow distinguishing between infected and vaccinated animals (DIVA strategy). Therefore, the generation of universal vaccines that induce effective protection against multiple virus serotypes is an increasingly pressing goal, especially since more than one BTV serotype circulates in all regions of the world where BTV is stably endemic.

Vaccines against BTV have commonly been aimed at the induction of broadly neutralizing antibody and T cell responses, since both arms of the adaptive immune response have a role in protection against BTV (12–14). The nonstructural (NS) proteins, NS1, NS2, NS3/3A, NS4, and the putative viral protein NS5, play a number of important roles in virulence, viral replication, maturation, and export, suggesting that NS proteins are candidate targets for antiviral therapies (15–18). NS1 is the most synthesized viral protein in BTV-infected cells and is highly conserved among different serotypes (16, 19–21). This protein contains epitopes associated with both T cell and humoral responses, and antibody responses against NS1 protein may be important contributors to immune protection (16, 22, 23).

The use of viral vaccine vectors, such as modified vaccinia Ankara virus (MVA), deployed in heterologous prime-boost regimes, has been routinely developed to induce strong T cell responses targeting intracellular pathogens (24). In fact, the heterologous prime-boost immunization using either DNA-MVA or muNSMi-microspheres-MVA expressing the structural proteins VP2 and VP7 confer total protection against heterologous challenges in the IFNAR^−/−^ mouse model when NS1 is included in the vaccine composition (25, 26). In this work we have analyzed the multiserotype protective capacity of protein NS1 when is delivered as a single antigen by a viral vector that induces a strong T cell immune response in this model. We have developed safe and DIVA experimental vaccines against BTV based on the recombinant viral vector MVA expressing NS1 or a truncated version of the protein (MVA-NS1-Nt).We show that cytotoxic CD8 T cell responses against NS1 provide essential help to confer protection against lethal challenge with several BTV serotypes in the absence of neutralizing antibodies. Furthermore, we demonstrate that the specific CD8 T cell epitope, NS1-152, is critical in order to elicit protection, since its deletion abolishes the protective capacity of this vaccine.

## RESULTS

### Evaluation of BTV-4 NS1, NS1-Nt, NS1-NtΔ152, and NS1-Ct expression from recombinant MVAs.

**FIG 1:**
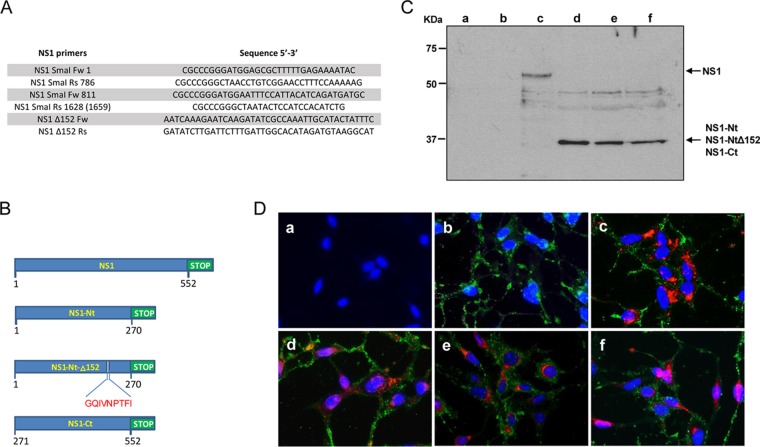
Table of primers and immunofluorescence of rMVAs. (A) Table of primers used to generate NS1-Ct, NS1-Nt, and NS1-NtΔ152. (B) Scheme of the NS1, NS1-Nt, and NS1-NtΔ152 constructions, with the deletion of p152 in NS1-NtΔ152. (C) Immunoblot analysis of NS1, NS1-Nt, NS1-Ct, and NS1-NtΔ152 in DF-1 cells mock infected (lane a) or infected with MVA-wt (lane b), MVA-NS1 (lane c), MVA-NS1-Nt (lane d), MVA-NS1-Ct (lane e), and MVA-NS1-NtΔ152 (lane f) at 24 h.p.i. using a mouse polyclonal serum specific for NS1. (D) Indirect immunofluorescence of DF-1 cells mock infected (a) or infected with MVA-wt (b), MVA-NS1 (c), MVA-NS1-Nt (d), MVA-NS1-Ct (e), and MVA-NS1-NtΔ152 (f) at 24 h.p.i. using a mouse polyclonal serum specific for BTV-16 (red) and a sheep polyclonal serum specific for MVA (green).

In order to evaluate the expression of NS1, NS1-Nt, NS1-NtΔ152, and NS1-Ct of BTV-4 (Fig. 1B) from recombinant MVA (rMVA) vectors in infected DF-1 cells, studies using immunoblot and immunofluorescence microscopy (IFA) were performed. Expression of NS1, NS1-Nt, NS1-NtΔ152, and NS1-Ct proteins with the expected molecular mass was observed at 24 h postinfection (Fig. 1C, lanes c to f), while no expression was detected in noninfected or MVA-wt-infected cells (Fig. 1C, lanes a and b). Immunofluorescence assays confirmed the expression of NS1, NS1-Nt, NS1-NtΔ152, and NS1-Ct in cells infected with the rMVAs (Fig. 1D). These data confirm the efficient expression of the proteins from BTV-4 cloned in the MVA vaccine vectors used for immunization of IFNAR^−/−^ mice.

### MVA-NS1 recombinant vaccine protects against challenge with multiple BTV serotypes.

**FIG 2:**
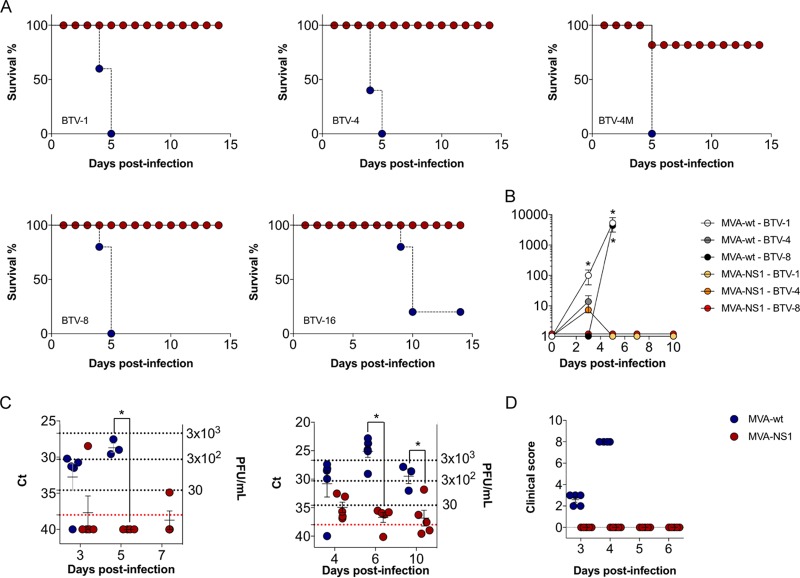
MVA-NS1 confers protection against multiple serotypes of BTV in IFNAR^−/−^ mice. (A) Survival rate after infection. Animals were inoculated with 10^7^ PFU of MVA-NS1 or MVA-wt as a negative control following a prime-boost strategy. Afterwards, animals were challenged with a lethal dose of different BTV serotypes. Red circles and continuous line, MVA-NS1-immunized mice; blue circle and dashed line, MVA-wt (B) Viral titers of BTV-1, BTV-4, and BTV-8 recovered in blood of control and immunized IFNAR^−/−^ mice after challenge. Virus was extracted from blood and determined as described in Materials and Methods. Points indicate group means, and error bars show the SDs. (C) Detection of BTV-4 strain Morocco (BTV-4M) (left) and BTV-16 (right) in blood of control and immunized IFNAR^−/−^ mice after challenge by RT-qPCR. Total RNA from blood and the expression of mRNA of segment 5 (encoding NS1 protein) were quantified at days 3, 5, and 7 postinfection for BTV-4M and 4, 6, and 10 postinfection for BTV-16. Results are expressed as *C_T_* values and PFU equivalents. Red lines represent the cutoff described by Toussaint et al. (47). (D) Postchallenge sickness scores for control and immunized IFNAR^−/−^ mice challenged with BTV-4. Animals were evaluated and scored for the following individual signs: rough hair (absent = 0, slight = 1, and marked = 2), activity (normal = 0, slightly reduced = 1, reduced = 2, and severely reduced = 3), eye swelling (absent = 0, slight = 1, moderate = 2, and severe = 3), and temperature (normal = 0 and hypothermia = 3). The final score was the addition of each individual score. The minimum score was 0 for healthy; scores of 1 to11 indicated severity. Animals that reached 10 points were euthanized. Each score represents the value of a single animal. Asterisks indicate statistical significance calculated using the parametric unpaired Student's *t* test (*P* < 0.05).

Adult IFNAR^−/−^ mice were immunized with MVA-NS1 or MVA-wt (control) by intraperitoneal injection in a prime-boost regimen at 3-week intervals. Two weeks after the second immunization, the mice were challenged subcutaneously with lethal doses of several BTV serotypes, including BTV serotype 1 (ALG2006/01) (BTV-1), BTV serotype 4 (SPA2004/02) (BTV-4), BTV serotype 4 strain Morocco (MOR2009/09) (BTV-4M), BTV serotype 8 (BEL/2006) (BTV-8), and BTV serotype 16 (RSArrrr/16) (BTV-16). Survival, viremia, clinical signs, and hematological parameters were then analyzed. After 5 days, all control animals had succumbed to infection, independently of serotype, except for one mouse that survived BTV-16 infection. In contrast, all MVA-NS1-immunized mice survived infection, except one mouse challenged with BTV-4M that died at 5 days postinfection (d.p.i.) (Fig. 2A).

We determined viremia after immunization and challenge by blood virus isolation in cell culture (for BTV-1, BTV-4, and BTV-8) and by reverse transcription-quantitative PCR (RT-qPCR) (for BTV-4M and BTV-16 due to the inability to these serotypes to form lysis plaques after virus blood recovery). Viremia was detected at 3 d.p.i., increasing thereafter until sacrifice in control infected animals, with titers up to 9.3 × 10^3^, 33, and 5 × 10^3^ PFU/ml by plaque assay for BTV-1, BTV-4, and BTV-8, respectively (Fig. 2B). In contrast, MVA-NS1-immunized mice did not show viremia by plaque assay after challenge (except one immunized mouse challenged with BTV-4 that showed reduced viremia at 3 d.p.i. returned to negativity for the next days of the experiment). We also analyzed by RT-qPCR the presence of BTV genomes in the blood of MVA-NS1-immunized and control IFNAR^−/−^ mice challenged with BTV-4M and BTV-16. BTV-4M genomes were readily detected in control mice at day 3 of infection (threshold cycle [*C_T_*] value mean: 32.7) and increased (*C_T_* value mean: 28.7) thereafter until sacrifice. In contrast, the RT-qPCRs yielded negative results for the majority of the MVA-NS1 immunized mice at all analyzed days postchallenge (*C_T_* value ≥ 38), except one immunized mouse that died (*C_T_* value mean: 28.4) and other that survived the challenge (*C_T_* value: 34.89) (Fig. 2C, left). For BTV-16 infection, we detected BTV-16 genomes at 4 d.p.i. (*C_T_* value mean: 30.8), with the highest level of BTV-16 genomes at day 6 postinfection (*C_T_* value mean: 25.08) and decreasing at 10 d.p.i. (*C_T_* value mean: 29.5), before the death of the animals in the control group. However, animals immunized with MVA-NS1 had negative or low viremia, reverting to negativity during the course of the infection (*C_T_* value means of 34.9, 36.7, and 36.8 for 4, 6, and 10 d.p.i., respectively) (Fig. 2C, right).

All control infected mice presented clinical signs (Fig. 2D). These were similar for all BTV serotypes, presenting rough hair coat, lethargy, eye swelling, and hypothermia, as opposed to the MVA-NS1-immunized mice, in which clinical signs were not detected. In the case of control group challenged with BTV-16, some animals developed hind leg paralysis at 10 d.p.i.

**FIG 3:**
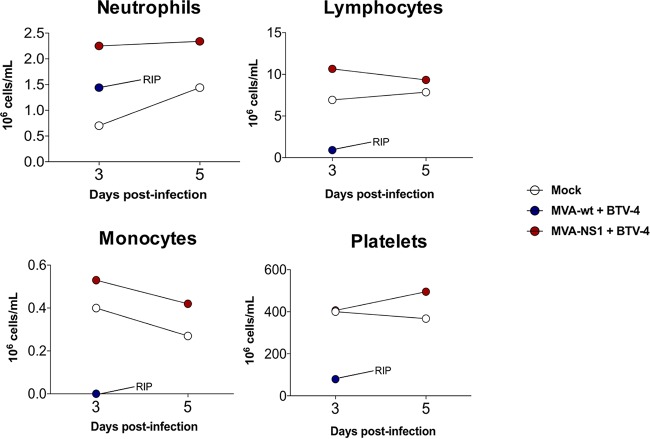
Hematological parameters in pooled blood of control and MVA-NS1-immunized IFNAR^−/−^ mice infected with BTV-4. An autohematology analyzer (BC-5300 Vet; Mindray, China) was used in these experiments. Neutrophils, lymphocytes, monocytes, and platelets were analyzed at days 3 and 5 postinfection. Pooled blood of nonimmunized and noninfected animals (mock) were used for reference values.

**FIG 4:**
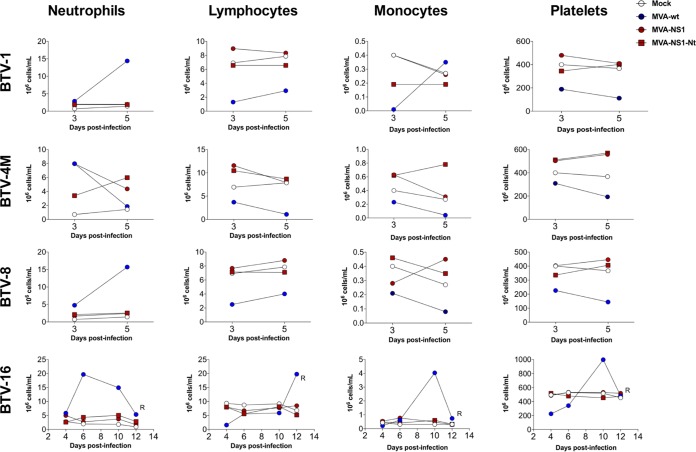
Blood parameters in MVA-NS1- and MVA-NS1-Nt-immunized IFNAR^−/−^ mice infected with BTV-1, BTV-4M, BTV-8, and BTV-16. An autohematology analyzer (BC-5300 Vet; Mindray, China) was used in these experiments. Neutrophils, lymphocytes, monocytes, and platelets were analyzed at days 3 and 5 postinfection for BTV-1, BTV-4M, BTV-8, and 4, 6, 10, and 12 for BTV-16. Pooled blood of nonimmunized and noninfected animals (mock) were used for reference values. Values for control infected animals (blue circles), MVA-NS1-immunized and infected animals (red circles), and MVA-NS1-Nt-immunized and infected animals (red squares) are shown.

We then determined hematological changes in mice after BTV infection. We evaluated pooled blood levels of neutrophils, lymphocytes, monocytes, and platelets in infected mice at 3 and 5 d.p.i. for BTV-1, BTV-4, BTV-4M, and BTV-8 and at 4, 6, 8, and 12 d.p.i. for BTV-16. BTV-4 infection resulted in a 10-fold drop in the absolute lymphocyte count, accompanied by a significant decrease in the levels of monocytes as well as a 5-fold decrease in the number of platelets (Fig. 3). These results were similar irrespective of the BTV serotype used for the challenge with few exceptions, such as the increase in the platelet counts in control animals infected with BTV-16 at 10 d.p.i., possibly due to coagulation disorders (Fig. 4). No significant differences were observed in neutrophil counts in BTV-4-infected control mice, possibly due to the faster pathology of this serotype (Fig. 3). In contrast, a significant increase was observed in BTV-1-infected, BTV-8-infected, and control BTV-16-infected animals (Fig. 4). MVA-NS1-immunized animals did not show variation in neutrophil levels after challenge, except those infected with BTV-4M, where an increase in the level of neutrophils was observed at 3 d.p.i., although this value returned to normal at 5 d.p.i. (Fig. 4).

All these data indicate that the immunization of mice with MVA-NS1 confers protection against multiple BTV serotypes and reduces or abrogates viremia and clinical signs while maintaining normal blood parameters.

### MVA-NS1 immunization generates strong CD8 T cell and nonneutralizing antibody responses.

**FIG 5:**
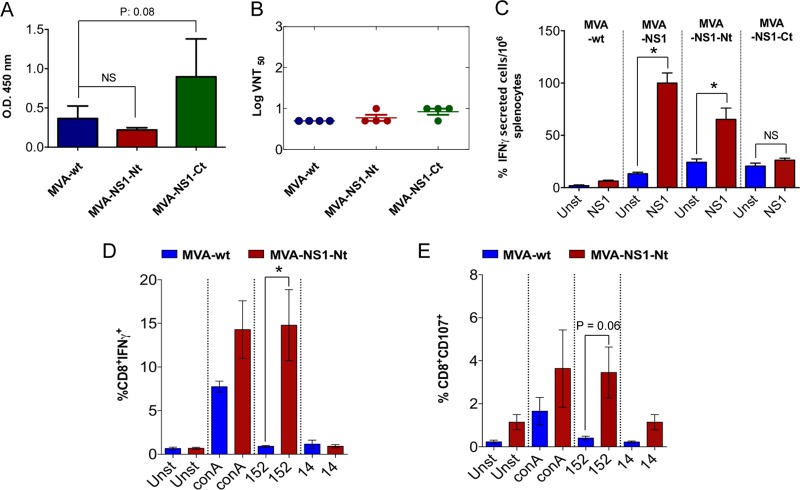
Immunogenicity of MVA-NS1-Nt and MVA-NS1-Ct recombinant viral vectors. (A) Analysis of the presence of antibodies specific for NS1 in serum of control MVA-wt and immunized MVA-NS1-Nt or MVA-NS1-Ct IFNAR^−/−^ mice by ELISA. Serum of immunized mice was collected 10 days postboost, and a dilution of 1:50 was analyzed by ELISA as described in Materials and Methods. (B) BTV-4 neutralizing antibody detection in MVA-wt-, MVA-NS1-Nt-, or MVA-NS1-Ct-immunized mice by VNT. Shown are neutralization titers in sera 10 days postboost. SDs are shown by error bars. Asterisks indicate statistical significance calculated using the nonparametric Mann-Whitney test (*P* < 0.05). NS, nonsignificant. (C) ELISPOT assays measuring IFN-γ-secreting T cells in the spleens of control (MVA-wt-immunized) and MVA-NS1-, MVA-NS1-Nt-, and MVA-NS1-Ct-immunized IFNAR^−/−^ mice. Splenocytes were harvested at day 10 postboost. Blue (unstimulated [unst]) and red (stimulated [NS1]) bars represent the mean number of spot-forming cells (SFCs) for the ELISPOT within each group. Error bars show SDs. A total of 10 μg/ml of recombinant NS1 per well was used as a stimulus in each experiment. (D and E) Intracellular staining of IFN-γ (D) or CD107a (E) in CD8 T cells of MVA-NS1-Nt-immunized animals. At 24 h poststimulation, intracellular IFN-γ production was analyzed in CD8 T cells, and at 6 h poststimulation, the indirect marker of cytotoxicity CD107a was also measured in CD8 T cells by flow cytometry. The results represent the averages from 4 mice ± SDs. Asterisks represent significant difference between samples, calculated by the Mann-Whitney nonparametric test (*P* < 0.05).

**FIG 6:**
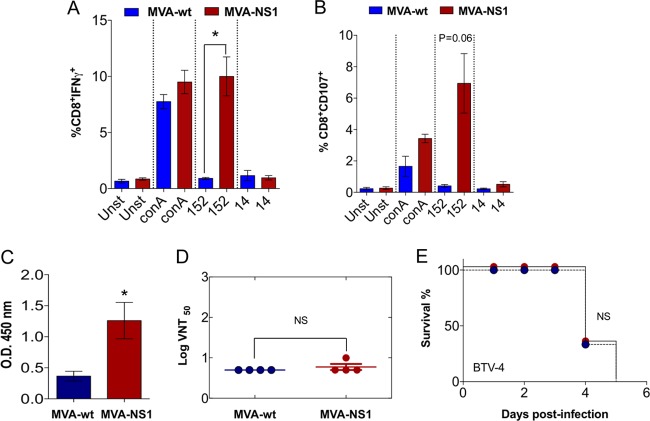
Immunogenicity of MVA-NS1 recombinant viral vector. Intracellular staining of IFN-γ (A) or CD107a (B) in CD8 T cells of MVA-NS1-immunized animals is shown. Ten days after the second immunization, spleens were harvested and the splenocytes were stimulated with NS1-152 peptide, using concanavalin A as a nonspecific stimulus, NS1-14 peptide as an irrelevant peptide, and RPMI 1640 as a negative control (unstimulated). At 24 h poststimulation, intracellular IFN-γ production was analyzed in CD8 T cells, and at 6 h poststimulation, the indirect marker of cytotoxicity, CD107a, was also measured in CD8 T cells by flow cytometry. (C) Analysis of the presence of antibodies specific of NS1 in serum of MVA-wt and MVA-NS1 IFNAR^−/−^ mice by ELISA. Serum of immunized mice was collected 10 days postboost, and a dilution of 1:50 was analyzed by ELISA as described in Materials and Methods. (D) BTV-4 neutralizing antibody detection in MVA-wt- and MVA-NS1-immunized mice by VNT. Neutralization titers in sera of control and immunized animals 10 days postboost are shown by blue circles (MVA-wt) and red circles (MVA-NS1). The results represent the averages from 4 mice ± SDs, shown as error bars. Asterisks indicate statistical significance calculated using the nonparametric Mann-Whitney test (*P* < 0.05). (E) Passive serum transfer. A total of 200 μl of pooled sera from MVA-NS1-immunized or MVA-wt animals was transferred intraperitoneally into naive IFNAR^−/−^ mice, and mice were challenged with a lethal dose of BTV-4 subcutaneously. Survival was analyzed during the experiment. Log rank (Mantel-Cox) test was used to compare groups.

In order to analyze the specific immune responses induced in mice immunized with MVA-NS1 we performed enzyme-linked immunosorbent spot (ELISPOT) assays, intracellular cytokine staining (ICS), virus neutralization tests (VNT), and enzyme-linked immunosorbent assays (ELISAs). Restimulation of splenocytes with recombinant NS1 protein yielded detectable specific gamma interferon (IFN-γ)-producing cells (mean spots: 100) in MVA-NS1-immunized mice but not in control animals (mean: 6.3) (Fig. 5C). We have recently determined that the 9-mer peptide GQIVNPTFI (i.e., p152) is an immunodominant CD8 T cell epitope from NS1. We restimulated splenocytes with peptide p152 and a nonrelevant peptide (p14) and determined by ICS IFN-γ production as well as CD107 cytotoxic marker expression in CD8 T cells. We observed the induction of CD8^+^ IFN-γ^+^ and CD8^+^ CD107^+^ cells upon restimulation of MVA-NS1 immunized mouse splenocytes with p152 (Fig. 6A and B). In contrast, the restimulation of splenocytes from control MVA-wt-immunized mice showed negligible responses to the peptide (Fig. 6A and B). These data confirmed that immunization with MVA-NS1 elicits a cytotoxic CD8 T cell response in mice, including to the immunodominant peptide p152.

We next performed an ELISA to detect NS1-specific antibodies in the sera of the immunized animals. Significant levels of NS1-specific antibodies were detected in MVA-NS1-immunized mice (optical density [OD] mean: 1.26) (Fig. 6C). Since commercially available diagnostics test for BTV are based on positive serology against the structural protein VP7, these results suggest that an ELISA diagnosis system based on detection of anti-NS1 antibodies could be an appropriate tool to discern between infected and MVA-NS1-vaccinated animals. In order to determine whether immunization with MVA-NS1 induced also the production of neutralizing antibodies, we performed a VNT. Although NS1 induced high levels of antibodies compared with the control, only negligible levels of neutralizing antibodies were detected in the sera of both immunized and control animals (Fig. 6D). To check whether anti-NS1 serum is involved in protection, heat-inactivated sera of control and immunized animals were also transferred intraperitoneally into naive mice (200 μl/animal) and challenged after serum transfer. All animals died (between 4 and 5 d.p.i.), and nonsignificant differences were observed between these groups (Fig. 6E).

Overall, our results show that a recombinant MVA-NS1 vaccine induces a potent and protective CD8 T cell immune response in the absence of neutralizing antibodies that are nevertheless amenable to be used as a tool to distinguish vaccinated and infected animals.

### The N- and C-terminal regions of NS1 elicit distinct immune responses.

According to the Immune Epitope Database (IEDB) (Kolaskar and Tongaonkar antigenicity results), the most antigenic residues of NS1 are located in the C-proximal region (NS1-Ct), while the amino-proximal region (NS1-Nt) contains mainly hydrophobic residues. We have reported that the most probable theoretical CD8 T cell-specific NS1 epitopes are found in the NS1-Nt region (25). We generated recombinant MVAs expressing NS1-Nt (MVA-NS1-Nt) and NS1-Ct (MVA-NS1-Ct). Mice were immunized with MVA-NS1-Nt, MVA-NS1-Ct, or MVA-wt (control), and antigen-specific immune responses were assayed by ELISA, VNT, and ELISPOT assay. Sera from immunized mice were analyzed for the presence of specific IgG antibodies against NS1. High levels of antibodies were observed in sera of MVA-NS1-Ct-immunized mice (mean OD: 0.89) (Fig. 5A). In contrast, sera from mice immunized with MVA-NS1-Nt or MVA-wt showed low antigen-specific antibody levels (mean OD: 0.22 and 0.36, respectively). These results support the *in silico* analysis, where NS1-Ct encompasses the most antigenic region of NS1. A VNT was also performed, confirming the absence of neutralizing antibodies (Fig. 5B). To further analyze the cellular immune response elicited by these fragments, the amount of IFN-γ produced by the cells after recombinant NS1 protein stimulation was determined by ELISPOT assay. After stimulation, MVA-NS1-Nt immunized mice developed detectable specific IFN-γ-producing cells (mean spots: 65.3), compared to the MVA-NS1-Ct-immunized and control splenocytes (means: 23.3 and 6.3, respectively) (Fig. 5C). The increase in the level of IFN-γ-producing cells after immunization with MVA-NS1-Nt was found to be significant when was analyzed by Student's *t* test compared with the control mice, but not when using MVA-NS1-Ct as an inmunogen. Furthermore, the peptide p152 was found to have induced a significant response in CD8 T cells by intracellular determination of IFN-γ production and the presence of the cytotoxic marker CD107 (Fig. 5D and E) in splenic cells from MVA-NS1-Nt-immunized mice, while no response was detected in splenocytes from control animals. These data suggest that the strategy of immunization based on MVA-NS1-Nt achieves response levels similar to those of MVA-NS1 and elicits an immune T CD8 cellular response in the animal model. These results also show that MVA-NS1-Ct elicits a potent, albeit nonneutralizing, humoral immune response, whereas MVA-NS1-Nt promotes the activation of cytotoxic CD8 T cells, in part through the peptide p152.

### NS1-Nt immunization mimics the multiserotype protective effect of NS1 that is dependent on p152.

**FIG 7:**
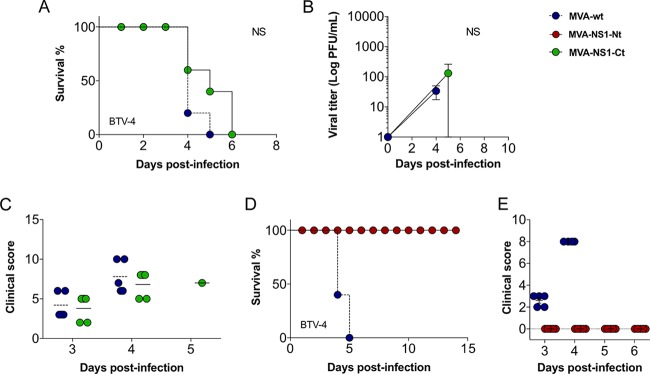
MVA-NS1-Nt protects against BTV-4, while MVA-NS1-Ct fails to confer protection in IFNAR^−/−^ mice. (A and D) Survival rates of mice immunized with MVA-NS1-Ct (A) and MVA-NS1-Nt (D) after infection with a lethal dose of BTV-4. Animals were inoculated with 10^7^ PFU following a prime-boost strategy. (B) Viral titers of BTV-4 recovered in blood from control and MVA-NS1-Ct-immunized IFNAR^−/−^ mice after challenge. (C and E) Postchallenge sickness scores of mice immunized with MVA-NS1-Ct (C) and MVA-NS1-Nt (E).

**FIG 8:**
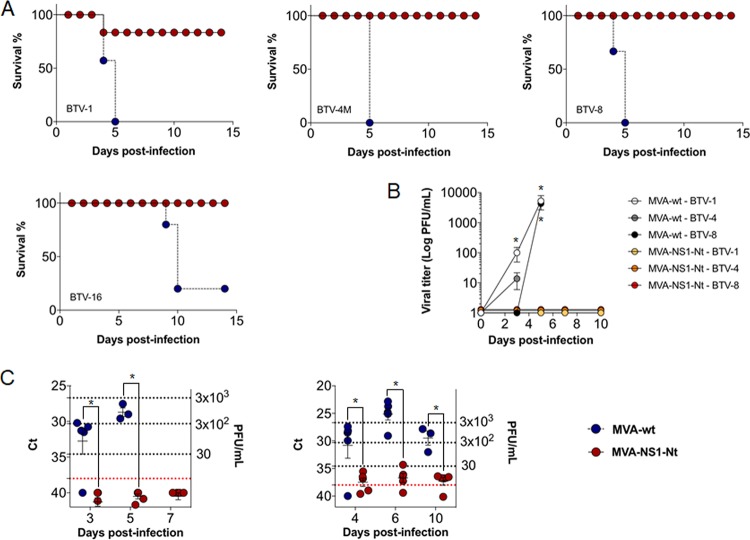
MVA-NS1-Nt confers protection against multiple serotypes of BTV in IFNAR^−/−^ mice. (A) Survival rate after infection. MVA-wt-immunized animals (control) and MVA-NS1-Nt-immunized animals were challenged against BTV-1, BTV-4M, BTV-8, and BTV-16. Control animals, blue circles; immunized animals, red circles. (B) Viral titers of BTV-1, BTV-4, and BTV-8 recovered in blood of control and MVA-NS1-Nt-immunized IFNAR^−/−^ mice after challenge. Virus was extracted from blood and determined as described in Materials and Methods. Points indicate group means, and error bars show SDs. (C) Detection of BTV-4 strain Morocco (BTV-4M) (left) and BTV-16 (right) in blood of control and immunized IFNAR^−/−^ mice after challenge by RT-qPCR. Total RNA from blood, and the expression of mRNA of segment 5 (encoding NS1 protein), was quantified at days 3, 5, and 7 postinfection. Results are expressed as *C_T_* and PFU equivalents. Red lines represent the cutoff described by Toussaint et al. (47).

**FIG 9:**
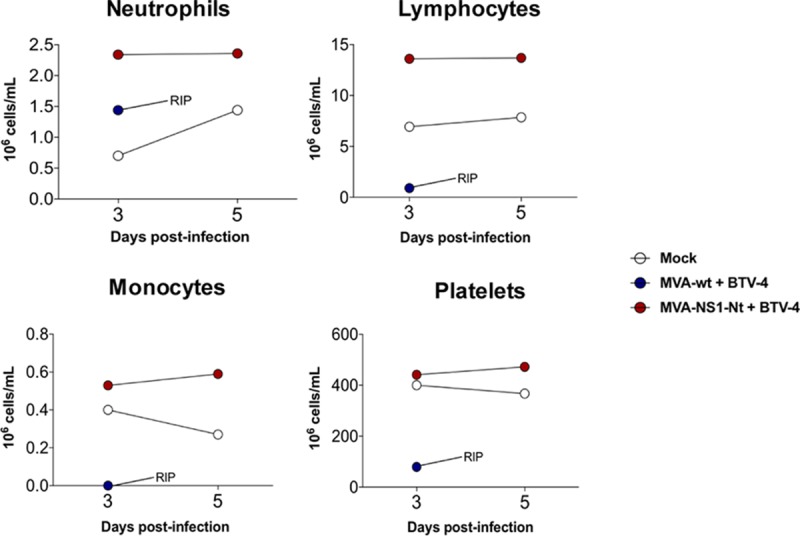
Hematological parameters in pooled blood of control and MVA-NS1-Nt-immunized IFNAR^−/−^ mice infected with BTV-4. An autohematology analyzer (BC-5300 Vet; Mindray, China) was used in these experiments. Neutrophils, lymphocytes, monocytes, and platelets were analyzed at days 3 and 5 postinfection. Blood of nonimmunized and noninfected animals (mock) were used for reference values. Asterisks indicate statistical significance calculated using parametric unpaired Student's *t* test (*P* < 0.05).

We then determined the immunoprotective capacity of immunization regimes with both MVA-NS1-Nt and MVA-NS1-Ct. Mice were immunized as described above with MVA-NS1-Nt and MVA-NS1-Ct and challenged with a lethal dose of BTV-4. A delay, albeit not significant (*P* = 0.11), in the mortality of animals immunized with MVA-NS1-Ct was observed compared with the control group (Fig. 7A). Some animals developed viremia and presented clinical signs (Fig. 7B and C). In contrast, all mice immunized with MVA-NS1-Nt were protected during the infection (Fig. 7D), in the absence of viremia (Fig. 8B) and clinical signs (Fig. 7E). When we evaluated the potential use of MVA-NS1-Nt as a multiserotype vaccine, we observed that all MVA-NS1-Nt-immunized mice survived BTV infections regardless of their serotype, except for one mouse challenged with BTV-1 that died at 4 d.p.i. (Fig. 8A). No viremia was detected in the blood of the immunized animals in the case of BTV-1, BTV-4, and BTV-8 by plaque assay (Fig. 8B), and negative or reduced RNA levels were found, respectively, in the case of BTV-4M and BTV-16 by RT-qPCR (Fig. 8C, left and right, respectively). The measurement of blood levels of neutrophils, lymphocytes, monocytes, and platelets at 3 and 5 d.p.i. showed that all these parameters remained within standard levels in the MVA-NS1-Nt-immunized animals for all the infections (Fig. 4 and 9). These data indicate that immunization with MVA-NS1-Nt confers protective immunity against multiple BTV serotypes, almost completely abrogating viremia and while maintaining normal blood parameters, mimicking immunization with the full-length NS1 protein.

**FIG 10:**
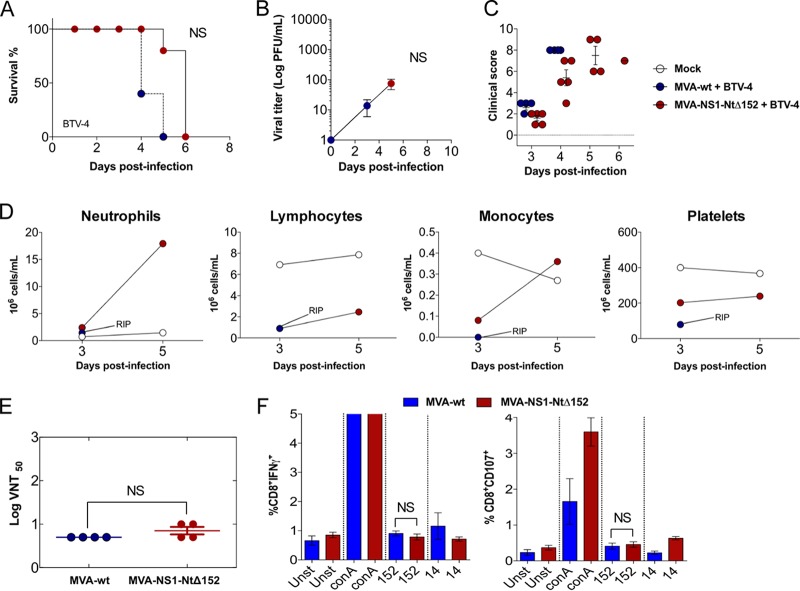
MVA-NS1-NtΔ152 loses its protection capacity against BTV-4 in IFNAR^−/−^ mice. (A) Survival rate after infection. Animals were inoculated with 10^7^ PFU of MVA-NS1-NtΔ152 or MVA-wt as a negative control following a prime-boost strategy and challenged with a lethal dose of BTV-4. (B) Viral titers of BTV-4 recovered in blood of MVA-wt- (control) and MVA-NS1-NtΔ152-immunized IFNAR^−/−^ mice after challenge. Each point represents the mean among the individual values of the viral titer of each animal, and SDs are shown as bars. (C) Postchallenge sickness score in control and MVA-NS1-NtΔ152-immunized IFNAR^−/−^ mice challenged with BTV-4. (D) Blood parameters in control and MVA-NS1-NtΔ152-immunized IFNAR^−/−^ mice infected with BTV-4. An autohematology analyzer (BC-5300 Vet; Mindray, China) was used in these experiments. Neutrophils, lymphocytes, monocytes, and platelets were analyzed at days 3 and 5 postinfection in pooled blood. Blood of noninfected and nonimmunized animals (mock) was used for reference values. (E) BTV-4 neutralizing antibody detection in control and MVA-NS1-NtΔ152-immunized mice by VNT. Neutralization titers in sera of immunized and control animals 10 days postboost are shown. SDs are shown as error bars. Asterisks indicate statistical significance calculated using the nonparametric Mann-Whitney test (*P* < 0.05). (F) Intracellular staining of IFN-γ (left) or CD107a (right) in CD8 T cells of MVA-NS1-NtΔ152 immunized animals. 10 days after the second immunization, spleens were harvested and the splenocytes were stimulated with NS1-152 peptide, using concanavalin A as a nonspecific stimulus, NS1-14 peptide as an irrelevant peptide, and RPMI 1640 as a negative control (unstimulated). At 24 h poststimulation, intracellular IFN-γ production was analyzed in CD8 T cells and at 6 h poststimulation, the indirect marker of cytotoxicity CD107a was also measured in CD8 T cells by flow cytometry. The results represent the averages from 4 mice ± SDs.

In order to address the role of peptide p152 in the immunoprotection elicited by NS1-Nt, we generated a recombinant MVA expressing a p152 deletion NS1-Nt mutant (MVA-NS1-NtΔ152). We then evaluated survival, viremia, and clinical signs upon BTV-4 challenge in animals immunized with this viral vector. No effect on the mortality of mice immunized with MVA-NS1-NtΔ152 was observed compared to the control group (*P* = 1.00) (Fig. 10A). Viremia was detectable at days 3 and 5 postchallenge (Fig. 10B) even though there was a delay in the onset of clinical signs (Fig. 10C). There was a decrease in lymphocytes, monocytes, and platelets and an increase in neutrophils in animals immunized with MVA-NS1-NtΔ152 and infected with BTV-4, a typical feature of control infected individuals (Fig. 10D). Immunization with MVA-NS1-NtΔ152 did not result in the production of neutralizing antibodies as was expected (Fig. 10E), and no induction of IFN-γ or surface CD107 was observed in CD8 T cells upon restimulation of splenic cells from MVA-NS1-NtΔ152-immunized mice with p152 (Fig. 10F).

These data demonstrate that within the N terminus of NS1, the immunodominant peptide p152 is required for full protection against BTV challenge.

## DISCUSSION

Protection against viral infection depends on the action of several immune effector mechanisms. For example, the presence of a strong type I interferon response is essential to blunt the initiation of infection, as demonstrated in strains of mice that are able to signal through IFNAR (27). For vaccine development, targeting the appropriate acquired immune response is critical for a successful protective effect. In the case of bluetongue, the generation of strong neutralizing antibodies against outer capsid antigens, such as VP2, provides full protection (28–35). Protection mediated by neutralizing antibodies has also been demonstrated for other orbiviruses, such as African horse sickness virus (36), and other emerging viral infections, including Zika, Ebola, Rift Valley fever, and yellow fever viruses (37–40). However, because surface antigens are highly variable among serotypes, vaccines based on these proteins are specific to the serotype they target and provide a poor cross-protection capacity. In order to confer multiserotype protection against BTV, CD8 T cell responses are required, probably because the antigens at which they are directed are highly conserved among serotypes (1, 22, 41, 42). Here we demonstrate the multiserotype protective capacity of MVA-based vaccines expressing the nonstructural protein NS1. Immunization with MVA-NS1 leads to high antibody titers with no neutralizing activity that are not able to induce protection when passively transferred to naive mice before challenge with BTV. Furthermore, the level of protection and induction of cytotoxic CD8 T cell activity is mimicked by the N-terminal domain of NS1 (NS1-Nt) in the absence of neutralizing antibodies. Remarkably, we pinpoint the protective capacity of this antigen to the presence of a CD8 T cell specific epitope within NS1-Nt.

NS1 is one of the major immunogens for CD8 T cells (1, 22). The primary antigenic site of NS1 has been localized within the carboxyl terminus, NS1-Ct (43); however, we identified the majority of theoretical CD8 T cell epitopes within the amino terminus of the protein (NS1-Nt). Indeed, our results show that while a recombinant MVA containing NS1-Ct (MVA-NS1-Ct) is able to induce high antibodies titers, it does not induce significant levels of IFN-γ-secreting CD8 T cells. Importantly, the immunization with MVA-NS1-Ct does not confer protection against a lethal challenge, although it induces a delay in the mortality rate of the mice.

In contrast to NS1-Ct, immunization with MVA-NS1-Nt leads to survival upon infection against multiple serotypes of BTV. Immunization with MVA-NS1-Nt induces low antibody titers. However, immunized mice develop strong cytotoxic CD8 T cell responses against this antigen. Our data demonstrate that the protective capacity of the vaccine based on NS1-Nt is due to the presence of the epitope, p152. Indeed, the deletion of this CD8 T cell epitope almost completely abrogates the protective effect elicited by MVA-NS1-Nt, although a small delay in the infection also occurs in its absence. These results demonstrate that the amino-terminal region of NS1 (NS1-Nt) is sufficient to induce protection against multiserotype BTV challenge through the induction of cytotoxic CD8 T cells that is largely dependent on the presence of the epitope p152.

Bluetongue is an important livestock disease worldwide. In order to control BTV expansion, the development of an efficient multiserotype vaccine that allows the differentiation between infected and vaccinated animals while protecting against all serotypes is needed. Our results show that a single highly conserved antigen expressed in a MVA vector can provide significant multiserotype protection against BTV that is largely dependent of a single CD8 T cell epitope. Furthermore, immunization with either MVA-NS1 or MVA-NS1-Nt was able to prevent viremia after challenge of IFNAR^−/−^ mice with BTV. This reduction of viremia not only prevents the development of disease symptomatology in the immunized animals but may also reduce virus acquisition by Culicoides bites.

Although further studies of this vaccination strategy, including the assessment of whether it induces long-lasting protection in the natural host, will be necessary, these data reveal the importance of the nonstructural protein NS1 in CD8 T cell-mediated protection against multiple BTV serotypes, when vectorized as a recombinant MVA vaccine. Furthermore, an ELISA diagnosis system based on recombinant NS1 recognition, in combination with the commercial ELISA BTV diagnostic test based on the protein VP7, could be a good tool to discern between naturally infected and MVA-NS1-vaccinated animals. Overall, these data demonstrate that the development of vaccines that can induce strong CD8 T cell responses against BTV based on NS1, NS1-Nt, or peptide 152 could accomplish maximal protective efficacy. Since peptide p152 has been involved in ovine T cell immunity (23), our data warrant further vaccine efficacy experiments with ruminants.

## MATERIALS AND METHODS

### Cells and viruses.

Chicken embryo fibroblasts (DF-1) (ATCC; catalog no. CRL-12203) and Vero cells (ATCC; catalog no. CCL-81) were grown in Dulbecco′s modified Eagle′s medium (DMEM) supplemented with 2 mM glutamine, 10% heat-inactivated fetal bovine serum (FBS), and antibiotics. BTV serotype 1 (ALG2006/01) (BTV-1), BTV serotype 4 (SPA2004/02) (BTV-4), BTV serotype 4 strain Morocco (MOR2009/09) (BTV-4M), BTV serotype 8 (BEL/2006) (BTV-8), and BTV serotype 16 (RSArrrr/16) (BTV-16) were used in the experiments. BTV-1, BTV-4, BTV-8, and MVA virus stocks and titrations were performed as previously described (29). BTV-4M and BTV-16 titrations were performed by RT-qPCR as previously described (44). NS1 sequence alignment and the percentage of identity of all serotypes of BTV used in this experiment are shown in Fig. 1.

### Immunoblot analysis.

Infected and noninfected lysed cells were analyzed by gradient sodium dodecyl sulfate-polyacrylamide gel electrophoresis (10% polyacrylamide). The proteins were transferred to a nitrocellulose membrane with a Bio-Rad Mini Protean II electroblotting apparatus at 150 mA for 2 h in 25 mM Tris–192 mM glycine buffer (pH 8.3) containing 20% methanol. Membrane binding sites were blocked for 1 h with 5% dried skim milk in TBS (20 mM Tris-HCl [pH 7.5], 150 mM NaCl). The membranes were then incubated with a mouse polyclonal serum specific for protein NS1. Bound antibody was detected with horseradish peroxidase-conjugated rabbit anti-mouse antibody and the ECL detection system (Amersham Pharmacia Biotech).

### Indirect immunofluorescence microscopy.

Cells were plated on glass coverslips and infected. Infections were performed at a multiplicity of infection (MOI) of 1 PFU/cell at 37°C in DMEM containing 2% FCS. Free viruses were removed after 90 min, and the cells were maintained in DMEM containing 2% FCS. At 24 h postinfection (h.p.i.), the cells were washed with PBS and fixed by addition of 4% paraformaldehyde for 30 min at room temperature. Cells were incubated with a PBS–20% FCS diluent containing 0.2% saponin (Fluka, Biochemika, Germany) for 1 h at room temperature and incubated overnight at 4°C with mouse polyclonal serum specific for BTV-16 and sheep polyclonal serum specific for MVA. Alexa Fluor goat anti-mouse 594 and Alexa Fluor goat anti-sheep 488 (Life Technologies) were used as secondary antibodies. The coverslips were washed four times with PBS and one time with PBS–4′,6-diamidino-2-phenylindole (PBS-DAPI; 1:10,000), mounted on glass slides, and analyzed with an Olympus CKX41 microscope.

### Animals and immunizations.

IFN-α/ßR^o/o^ IFNAR^−/−^ 129/Sv mice were purchased from B&K Universal Ltd. (UK). Eight-week-old male mice were used throughout. Upon reception, the mice were held for 7 days for acclimatization under pathogen-free conditions in the biosafety level 3 (BSL3) animal facility at the Center for Animal Health Research (INIA-CISA), Madrid, Spain.

Groups of four or five IFNAR^−/−^ mice were immunized by homologous prime-boost vaccination with recombinant MVAs expressing NS1, NS1-Nt, NS1-Ct, and NS1-NtΔ152 variants of BTV-4 or wild-type MVA (MVA-wt) (control group), administered 3 weeks apart. A total of 10^7^ PFU of each rMVA construct, the lowest dose that provides total protection, was inoculated intraperitoneally. Blood samples were collected from the submandibular plexus (100 μl/mouse/punctum, approximately) to perform the analysis of viremia and blood parameters.

Animals were evaluated and scored for the following individual clinical signs: rough hair (absent = 0, slightly = 1, and marked = 2), activity (normal = 0, slightly reduced = 1, reduced = 2, and severely reduced = 3), eye swelling (absent = 0, slightly = 1, moderate = 2, and severe = 3), and temperature (normal = 0 and hypothermia = 3). The final score was the addition of each individual score. The minimum score was 0 for healthy and 1 to 11 depending upon the severity. Animals that reached 10 points of score were euthanized. Each score represents the value of a single animal.

### Generation of recombinant MVAs.

The generation of MVA-NS1 has been previously described (26, 45). To generate MVA-NS1-Nt and MVA-NS1-Ct, a primer set targeting the N-terminal region of NS1 (amino acids [aa] 1 to 270) (NS1 SmaI Fw 1 and NS1 SmaI Rs 786) and another primer set targeting the C-terminal region of NS1 (aa 271 to 543) (NS1 SmaI Fw 811 and NS1 SmaI Rs 1628) were used to construct the transfer vectors pSC11-NS1-Nt and pSC11-NS1-Ct from pSC11-NS1. A 152-deletion mutant NS1-Nt MVA (MVA-NS1-NtΔ152) was designed. To generate the deletion, oligonucleotide primers NS1 SmaI Fw 1 and NS1 Δ152 Rs, deleting 9 aa (GQIVNPTFI), were used to generate a PCR product from nucleotides (nt) 1 to 456 of the NS1 gene. The primers NS1 Δ152 Fw, including the deletion, and NS1 SmaI Rs 786 were used to generate a PCR product from nt 485 to 786 of the NS1 gene. Both overlapping PCR products were used as templates for PCR amplification using the primers NS1 SmaI Fw 1 and NS1 SmaI Rs 786. The amplified DNA was digested with SmaI and cloned into the SmaI-digested pSC11 to obtain the pSC11-NS1-NtΔ152. All the sequences of the primers used in are listed in Fig. 1A.

### Humoral immune response assays.

The virus neutralization test (VNT) was used to determine neutralizing antibody titers against BTV-4. For plaque reduction assays, 2-fold dilutions of sera were mixed with 100 PFU of BTV-4, incubated for 1 h at 37°C, and then plated into monolayers of Vero cells. After 1 h, agar overlays were added and the plates were incubated for 5 days. The titer was determined as the highest dilution that reduced the number of plaques by 50%.

Serum was analyzed for antibodies by ELISA as previously described (29). Recombinant NS1 protein was adsorbed to 96-well Nunc Immuno Maxisorp plates at a concentration of 150 ng/μl in carbonate-bicarbonate buffer. Briefly, plates were washed with PBS containing 0.05% Tween 20 (PBS-T) and blocked with 5% skim milk powder in PBS-T. Sera were diluted to 1:50, added in duplicate wells. Bound antibodies were detected using alkaline phosphatase-conjugated rabbit anti-mouse total IgG (Bio-Rad, USA). Plates were developed by adding 3,3′,5,5′-tetramethylbenzidine (TMB) substrate. Optical density (OD) was read at 450 nm.

### *Ex vivo* IFN-γ ELISPOT and flow cytometric analysis.

Groups of IFNAR^−/−^ mice (*n* = 4) were immunized following a homologous prime-boost regimen with rMVA-NS1, rMVA-NS1-Nt, rMVA-NS1-Cţ rMVA-NS1-NtΔ152, rMVA-152, or MVA-wt (control group) 3 weeks apart. All animals were sacrificed at 10 days postboost, and their spleens were harvested for analysis by ELISPOT and intracellular cytokine staining (ICS) as previously described (33, 46).

ELISPOT assays were performed with mouse IFN-γELISPOT Ready-SET-Go (eBioscience), according to the method recommended by the manufacturer. A total of 5 × 10^5^ splenocytes were added to the well and stimulated with 10 μg/ml of recombinant NS1 protein. Plates were incubated at 37°C and 5% CO_2_ for 18 to 20 h. As a positive control, phytohemagglutinin (PHA) was used. Plates were scanned on an ImmunoSpot reader (Cellular Technology Ltd.). Specific spots were counted using ImmunoSpot software. The threshold value to consider a positive response by ELISPOT assay was that the number of specific spots/well had to be at least 2 times the average values found in negative-control wells of each group, and that after subtraction of background values (MS protein-stimulated splenocytes). For the ICS assay, a total of 10^6^ splenocytes were stimulated with 10 μg/ml of NS1-152 peptide, concanavalin A as a nonspecific stimulus (4 μg/ml), and NS1-14 peptide as an irrelevant peptide (10 μg/ml) or left untreated for 18 h in RPMI 1640 supplemented with 10% FCS and including brefeldin A (5 μg/ml) to increase the accumulation of IFN-γ in the responding cells. After stimulation, cells were washed, stained for the surface markers, fixed, permeabilized with PBS–1% FBS formaldehyde–4% saponin–1% buffer, and stained intracellularly using the appropriate fluorochromes. To analyze the adaptive immune responses, the following fluorochrome-conjugated antibodies were used: anti-mouse CD8^−^ peridinin chlorophyll protein (PerCP)-cyanine 5.5, IFN-γ–phycoerythrin (PE), from eBioescience, and CD107a–LAMP-1–fluorescein isothiocyanate (FITC) from Miltenyi. Data were acquired by fluorescence-activated cell sorter (FACS) analysis on a FACSCalibur (Becton Dickinson). Analyses of the data were performed using FlowJo software version X0.7 (Tree Star, Ashland, OR). The number of lymphocyte-gated events was 5 × 10^5^.

### Adoptive transfer of serum.

Sera of MVA-NS1- and MVA-wt-immunized animals were collected and pooled, and 200-μl volumes were transferred intraperitoneally. Recipient mice were challenged subcutaneously with a lethal dose of BTV-4 simultaneously with the transfer. Viral titers in blood were measured by plaque assay, and clinical signs were also evaluated.

### *In vivo* infections.

Two weeks after immunization, mice were subcutaneously inoculated with 10^2^ PFU of BTV-1 or BTV-8, 5 ×10^2^ PFU of BTV-4, 10 PFU of BTV-4M, or 10^4^ PFU of BTV-16 (lethal doses). Mice were bled before each immunization and after virus challenge at 3, 5, 7, 10, and 15 d.p.i. or 4, 6, 10, and 12 d.p.i. for BTV-16. Sera were tested for BTV-4 neutralizing antibodies by a VNT and ELISA. Blood was collected at different times to study viremia and hematological parameters. Viremia was analyzed by plaque assay (for BTV-1, BTV-4, and BTV-8) or measured by real-time RT-qPCR specific for BTV segment 5 (for BTV-4M and BTV-16, unable to form lysis plaques in cell culture). The real-time RT-qPCR specific for BTV segment 5 was performed as described by Toussaint et al. (47), and mouse blood samples containing different concentrations of virus were titrated and used as standards.

### Blood measurements.

A multiparameter autohematology analyzer (BC-5300 Vet; Mindray, China) was used to determine the total and differential cell counts in pools of blood for each group and collected into EDTA tubes.

### Statistical analysis.

Data were analyzed using GraphPad Prism version 6.0 for Windows (GraphPad Software, San Diego, CA). Survival analysis was performed using log rank test. Parametric unpaired Student's *t* test was performed to compare mean responses between two groups for viremia analysis. Wilcoxon signed-rank test was performed to compare mean responses between two groups for VNT. ELISPOT and ICS analysis were performed using the Mann-Whitney nonparametric test. A *P* value of 0.05 was considered significant in all cases.

### Ethics statement.

Animal experimental protocols were approved by the Ethical Committee of the Center for Animal Health Research (CISA-INIA) (permit number PROEX 037/15) in strict accordance with Spanish National Royal Decree (RD1201/2005), international EU guidelines (2010/63/UE) regarding protection of animals used for experimentation and other scientific purposes, and Spanish Animal Welfare Act 32/2007. All work with infected animals was performed in a BSL3 laboratory of the Center for Animal Health Research (CISA-INIA).
